# The Elderly Patient with Atrial Fibrillation: Optimal Treatment Strategies

**DOI:** 10.3390/jcm14051753

**Published:** 2025-03-05

**Authors:** Fabiana Lucà, Iris Parrini

**Affiliations:** 1 Department of Cardiology, Grande Ospedale Metropolitano (GOM) of Reggio Calabria, Bianchi Melacrino Morelli Hospital, 89129 Reggio Calabria, Italy; 2 Department of Cardiology, Mauriziano Hospital, 10020 Turin, Italy; irisparrini@libero.it

Atrial fibrillation (AF) is the most common sustained cardiac arrhythmia, with its prevalence expected to rise significantly due to global population aging [1]. The condition is characterized by an irregular and often rapid heart rhythm, predisposing individuals to adverse outcomes such as stroke, heart failure, and cognitive decline. In the elderly population, managing AF is particularly challenging due to the interplay of multimorbidity, frailty, polypharmacy, and reduced physiological reserves [1]. This review investigates optimal strategies for managing AF in elderly patients, focusing on rate and rhythm control, anticoagulation, and preventing associated complications.

The susceptibility of elderly individuals to AF can be attributed to both structural and electrical remodeling of the heart. Aging leads to progressive atrial fibrosis, amyloid deposition, and dilation, impairing myocardial elasticity and increasing atrial pressures [2,3,4]. Electrical remodeling, including slowed conduction velocities and prolonged refractory periods, further predisposes to arrhythmogenic mechanisms [5,6], Chronic kidney disease (CKD) [7] has also been found to be highly prevalent in older adults and exacerbate the pathophysiological changes seen in AF.

Effective management of AF in elderly patients necessitates a comprehensive geriatric assessment (CGA) to evaluate a patient’s frailty, functional capacity, and overall health status. CGA tools such as the FRAIL scale, 5-meter gait speed test, Mini Nutritional Assessment (MNA), and Mini-Cog test are invaluable in identifying vulnerable individuals. Additional assessments, including sarcopenia evaluation and bioimpedance analysis, help stratify risk and guide therapeutic decisions. Incorporating these assessments allows clinicians to optimize treatment strategies while minimizing any potential harm from interventions.

Rate control is often the preferred strategy in elderly patients with long-standing AF, multiple comorbidities, or limited life expectancy. It aims to alleviate symptoms and prevent tachycardia-induced cardiomyopathy. The RACE II trial demonstrated that lenient rate control (≤110 bpm) was non-inferior to strict control (<80 bpm) in terms of cardiovascular outcomes.

In contrast, rhythm control may benefit symptomatic patients, particularly those with recent-onset AF, preserved functional capacity, or concomitant heart failure. The EAST-AFNET 4 trial highlighted the advantages of early rhythm control, including reduced cardiovascular hospitalizations and improved quality of life. However, in frail or multimorbid patients, rhythm control requires careful consideration due to the risks associated with antiarrhythmic drugs and invasive procedures.

Antiarrhythmic drugs (AADs) remain a cornerstone of rhythm control but require careful selection in elderly patients due to their side effect profiles [8]. Amiodarone, while effective, poses significant risks of thyroid, pulmonary, and hepatic toxicity, particularly with long-term use. Dronedarone offers a safer alternative but is contraindicated in patients with severe heart failure. Flecainide is suitable for patients with structurally normal hearts but should be avoided in those with coronary artery disease. Evidence from the ORBIT-AF [9] study underscores the importance of individualized AAD therapy in achieving optimal outcomes.

Catheter ablation (CA) has emerged as an effective option for AF management, particularly in symptomatic, drug-refractory cases. Studies such as CASTLE-HF [10] and CABANA [11] have demonstrated the benefits of ablation in reducing AF recurrence and improving the quality of life. However, the procedural risks are heightened in patients over 75 years, necessitating a careful risk–benefit analysis. For frail individuals, the decision to pursue ablation should be informed by patient preferences, life expectancy, and the presence of comorbidities [12,13] (Figure 1).

Stroke prevention through anticoagulation is a critical component of AF management in the elderly population. Direct oral anticoagulants (DOACs) have largely supplanted vitamin K antagonists (VKAs) due to their predictable pharmacokinetics, reduced bleeding risk, and lack of routine monitoring requirements [14]. Among DOACs, apixaban has demonstrated superior safety in frail elderly patients, with a lower incidence of major bleeding compared with rivaroxaban or dabigatran [15,16,17]. Regular monitoring of renal function and adherence is essential to ensure therapeutic efficacy and minimize complications [16].

Frailty is not a contraindication to anticoagulation; however, it necessitates careful assessment of bleeding risks using tools such as the HAS-BLED score [12]. Recent evidence suggests that anticoagulation, even in frail patients, significantly reduces the risk of thromboembolic events, without a proportional increase in major bleeding [12].

Emerging data indicate a strong association between AF and cognitive decline, mediated by mechanisms such as cerebral hypoperfusion, microinfarcts, and systemic inflammation. AF may accelerate the progression of vascular dementia and Alzheimer’s disease through impaired cerebral perfusion and increased amyloid deposition. Observational studies, such as SAGE-AF, suggest that rhythm control strategies may offer neuroprotective benefits by stabilizing the cerebral blood flow [12]. Optimal anticoagulation further mitigates the risk of silent cerebral infarctions, a contributor to cognitive decline in AF patients [12].

The management of AF in elderly patients is inherently complex, requiring a personalized approach that accounts for individual risk factors, comorbidities, and patient preferences [12]. Rate control remains the cornerstone for frail or multimorbid patients, while rhythm control may benefit younger or less frail individuals with symptomatic AF. Anticoagulation is indispensable for stroke prevention, with DOACs being preferred due to their favorable safety profile. Future research should prioritize randomized controlled trials focusing on the quality of life, cognitive outcomes, and the integration of geriatric assessments into AF management pathways (Figure 2).

## Figures and Tables

**Figure 1:**
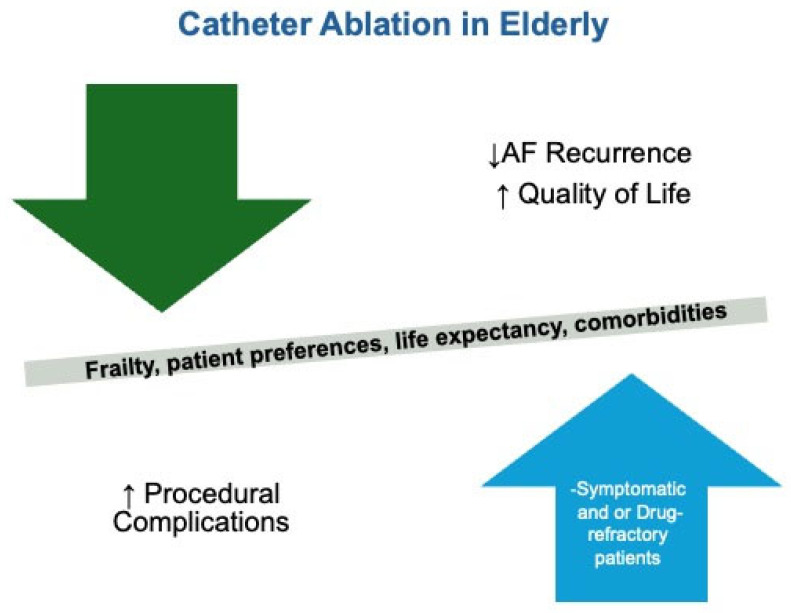
Catheter ablation in elderly patients.

**Figure 2:**
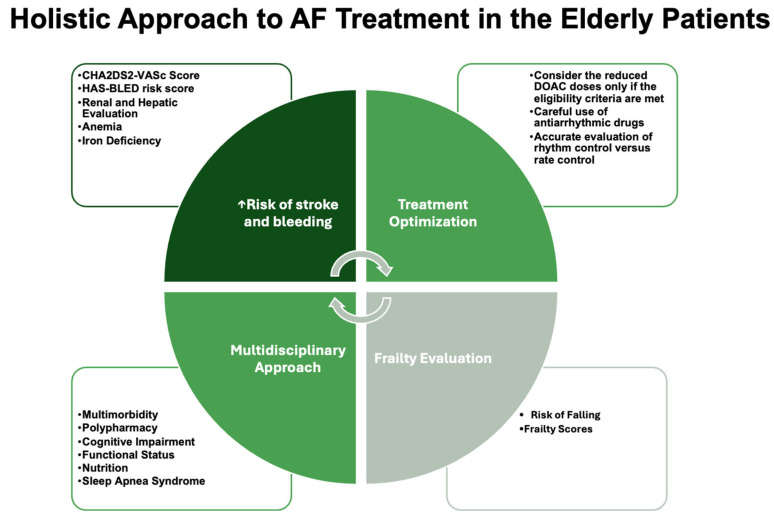
The role of a holistic approach to AF in elderly patients.
